# A comprehensive analysis of IDO1 expression with tumour‐infiltrating immune cells and mutation burden in gynaecologic and breast cancers

**DOI:** 10.1111/jcmm.15176

**Published:** 2020-03-30

**Authors:** Xu Feng, Ranran Tang, Runjie Zhang, Huan Wang, Zhaodong Ji, Yang Shao, Shuoer Wang, Tianying Zhong, Yun Gu, Jiao Meng

**Affiliations:** ^1^ Cancer Institute Fudan University Shanghai Cancer Center Shanghai China; ^2^ Department of Oncology Shanghai Medical College Fudan University Shanghai China; ^3^ Nanjing Maternity and Child Health Care Hospital Women's Hospital of Nanjing Medical University Nanjing China; ^4^ Central Laboratory The Fifth People's Hospital of Shanghai Fudan University Shanghai China

**Keywords:** breast cancers, gynaecologic cancers, IDO1, immunotherapy, TMB

## Abstract

Gynaecologic and breast cancers share some similarities at the molecular level. The aims of our study are to highlight the similarities and differences about IDO1, an important immune‐related gene in female cancers. The NGS data from TCGA of cervical squamous cell carcinoma (CESC), ovarian serous cystadenocarcinoma (OV), uterine corpus endometrial carcinoma (UCEC), uterine carcinosarcoma (UCS) and breast invasive carcinoma (BRCA) were analysed to identify molecular features, and clinically significant and potential therapeutic targets of IDO1. We found IDO1 was significantly up‐regulated in four gynaecologic cancers and breast cancer. According to breast cancer PAM50 classification scheme, IDO1 expression was higher in tumours of basal than other subtypes and showed better survival prognosis in BRCA and OV. Through immune infiltration analysis, we found a strong correlation between IDO1 and immune cell populations especially for dendritic cells and T cells. In addition, we investigated the association between IDO1 and tumour mutation burden (TMB) and found that IDO1 was significantly correlated with TMB in BRCA and CESC. GSVA revealed that hallmarks significantly correlated with IDO1 were involved in interferon gamma response, allograft rejection and inflammatory response. We also found PD‐L1 and LAG3 were highly positive related to IDO1 in gynaecologic cancers when comparing with their corresponding normal tissues. Our results indicated that IDO1 participated in anti‐tumour immune process and is correlated with mutation burden. These findings may expand our outlook of potential anti‐IDO1 treatments.

## 1 INTRODUCTION

Gynaecologic and breast cancers are considered as global public health problems. They share a variety of characteristics, such as similar embryonic origins in the Mullerian ducts and influenced by female hormones.^1^, ^2^ Gynaecologic and breast cancers have an estimated incidence/deaths of more than 773 000/237 700 and 380 270/75 360 cases in Europe (2018) and the United States (2019).^3^, ^4^ Breast cancer is the first and uterine corpus cancer is the fourth most common cancer in women.^4^ Therefore, it is necessary to discover and identify the novel and specific strategies to treat and prevent carcinogenesis of those cancers in women.

Cancer progression is a comprehensive process depending on the interaction between individual cells in the tumour, the microenvironment and the immune system, all of which can act to promote or suppress tumour growth and metastasis.^5^ Increasing evidence revealed that microenvironment plays a critical role in supporting the progression of tumours. Several studies reported an increased tumour‐infiltrating lymphocytes (TILs) in different tumours and correlations between specific immune subsets and prognosis.^6^, ^7^ Consequently, immune therapy alone or in combination with radiation and chemotherapeutic therapy is the current meaningful neoadjuvant treatment. Immuno‐antibodies that target immune check point such as CTLA‐4, PD1 and PD‐L1 have significantly improved survival in several tumours.^8^

Immune evasion is one of the hallmarks of cancer, and researchers clarified complicated mechanisms to enable cancer cells escaping from the host immune response. One of the mechanisms is that cancer cells utilize the indoleamine 2,3‐dioxygenase (IDO) pathway to suppress immune surveillance.^9^, ^10^, ^11^ IDO1 is a haeme‐containing enzyme which catalyses the breakdown of tryptophan to kynurenine. Try is an essential amino acid obtained from the diet that is a fuel required by the body to build proteins needed for cellular growth as well as immune function. In healthy status, IDO1 removes the extra fuel needed for immune activity, and it ensures the balance of immune system and acts to inhibit the immune response through the following mechanisms: (a) inhibition of effector T cell activity to suppress the immune response; and (b) promotion of T regulatory cell activity to suppress the immune response. Namely, elevated IDO activity leads the consumption of tryptophan in the tumour microenvironment. Without tryptophan to fuel the immune cells, cytotoxic T cells starve and immunosuppressive Tregs are up‐regulated leading to a failure of the immune system to respond appropriately to the cancer.^12^ Human IDO1 has an evolutionary paralog (indolamine‐2,3‐dioxygenase 2, IDO2) and a functional ortholog (tryptophan‐2,3‐dioxygenase, TDO2) that catalyse the same biochemical reaction. IDO1 also has high enzyme activity for Trp (Km ~20 μmol/L).^13^ The expression of IDO1 and IDO2 is restricted to eukaryotes, but IDO2 has almost 1000‐fold lower enzyme activity (Km ~6.8 mmol/L)^14^ compare to IDO1. TDO2 is a tetrameric haeme‐containing complex and conserved across different species including both prokaryotes and eukaryotes with lower enzyme activity for Trp (Km ~190 μmol/L)^15^ compared to IDO1.

The function of IDOs as a checkpoint used by tumours to escape immune surveillance was a focus of research and drug discovery efforts, as well as efforts to understand whether it could be used as a biomarker for prognosis. The aim of this paper was to provide a comprehensive analysis of IDOs in gynaecologic and breast cancers, especially focusing on the molecular features of IDOs in order to improve the efficacy of currently available immune therapeutic strategies.

## 2 METHODS

### 2.1 Expression analysis

Expression analysis of IDO1, IDO2 and TDO2 based on TCGA and GTEx samples was conducted in GEPIA (http://gepia.cancer%2010pku.cn/index.html).^16^ The data of IDO1 expression in TCGA BRCA subtypes were downloaded in UCSC Xena (http://xena.ucsc.edu) and analysed by R software and GraphPad.

### 2.2 Survival analysis

Overall survival (OS) and disease‐free survival (DFS) were analyzed via GEPIA database. GEPIA uses log‐rank test for the hypothesis evaluation. The Cox proportional hazard ratio is based on Cox PH model.

### 2.3 Gene signatures and ssGSEA

The marker gene set for immune cell types was obtained from Bindea et al.^17^ We used single‐sample gene set enrichment analysis (ssGSEA) in R package gsva to calculate gene set enrichment. The deconvolution approach used in our study included 24 immune cells, which were dendritic cells (DCs), activated dendritic cells (aDC), immature dendritic cells (iDC), plasmacytoid dendritic cells (pDC), B cells, CD8 T cells, cytotoxic cells, eosinophils, macrophages, mast cells, neutrophils, natural killer cells (NK cells), NK CD56bright cells, NK CD56dim cells, T cells, T central memory (Tcm) cells, T effector memory (Tem) cells, T follicular helper (TFH) cells, T gamma delta (Tgd) cells, T helper cells, T helper 1 (Th1) cells, Th2 cells, Th17 cells and regulatory T (TReg) cells.

### 2.4 Tumour mutation burden data

Tumour mutation burden (TMB) data for the TCGA cohort were obtained from cBioPortal.^18^ Tumour data were selected samples from TCGA projects which include breast invasive carcinoma (BRCA) and four projects to represent the gynaecologic cancers: endocervical adenocarcinoma (CESC), high‐grade serous ovarian cystadenocarcinoma (OV), uterine corpus endometrial carcinoma (UCEC) and uterine carcinosarcoma (UCS).

### 2.5 Statistical analysis

In this study, statistical analysis was mainly performed using R language (https://www.r-project.org/) with several publicly available packages (R x64 3.5.1). Multivariate survival analysis was performed by using Cox proportional hazards model. A probability value (*P*) <.05 was considered to be significant in this study.

## 3 RESULTS

### 3.1 IDO1 expression status and clinical outcome

**Figure 1:**
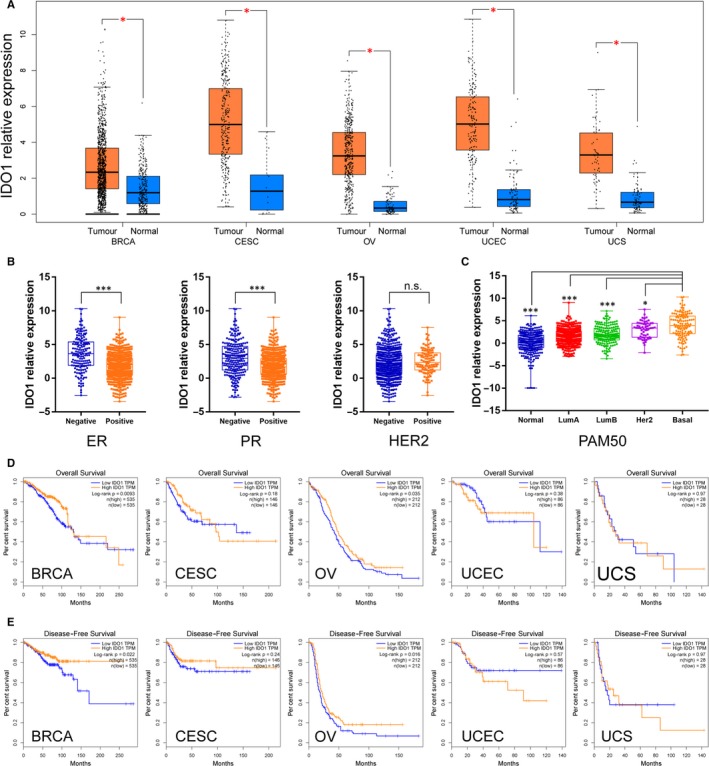
Landscape of IDO1 expression status and clinical outcome in gynaecologic and breast cancers. A, Comparison of IDO1 expression between gynaecologic and breast cancers (TGCA) and normal gynaecologic and breast tissues (GTEx). BRCA, breast invasive carcinoma; CESC, cervical squamous cell carcinoma and endocervical adenocarcinoma; OV, ovarian serous cystadenocarcinoma; UCEC, uterine corpus endometrial carcinoma; UCS, uterine carcinosarcoma. B, IDO1 expression according to ER, PR or HER2 status in BRCA. C, IDO1 expression according to PAM50 status in BRCA. D, Overall survival (OS) of gynaecologic and breast cancer patients with high and low IDO1 expression status. E, Disease‐free survival (DFS) of gynaecologic and breast cancer patients with high and low IDO1 expression status

Considering not only IDO1 but also IDO2 and TDO2 could catalyse the first and rate‐limiting step of tryptophan (Trp) catabolism, we analysed both of their expression status with GEPIA, a web‐based tool to deliver fast and customizable functionalities based on the cancer genome atlas (TCGA) and genotype‐tissue expression (GTEx) data. As shown in Figure 1A and Figure S1, higher IDO1 expression was found in both breast and gynaecologic cancers, while TDO2 was higher expressed in BRCA, OV and UCS. However, IDO2 had no significant difference between normal and cancer samples in breast and gynaecologic cancers (Figure S1). When taking breast cancer subtypes into account, we found that IDO1 was highly expressed in ER‐negative, PR‐negative and Her2‐negative samples (Figure 1B) and basal‐like breast cancer than other types breast cancers (Figure 1C). Then, we explored the prognostic value of IDO1 in breast and gynaecologic cancers. Kaplan‐Meier analysis demonstrated that higher IDO1 expression was associated with better overall survival and disease‐free survival in BRCA and OV (Figure 1D,E). Higher IDO2 expression was also associated with better OS in BRCA and CESC (Figure S2A), while its lower expression predicts a worse DFS in UCS (Figure S2B). These data suggest a complicated character of IDO2 in female cancer. TDO2 had no significant effect on OS and DFS in breast and gynaecologic cancers (Figure S2C,D). Based on the expression, clinical outcome results and the enzyme activity to catalyse tryptophan to kynurenine, IDO1 may act as a more important role in tumour, so we choose IDO1 as the protagonist of the follow‐up analysis.

### 3.2 IDO1 expression and tumour‐infiltrating immune cells

**Figure 2:**
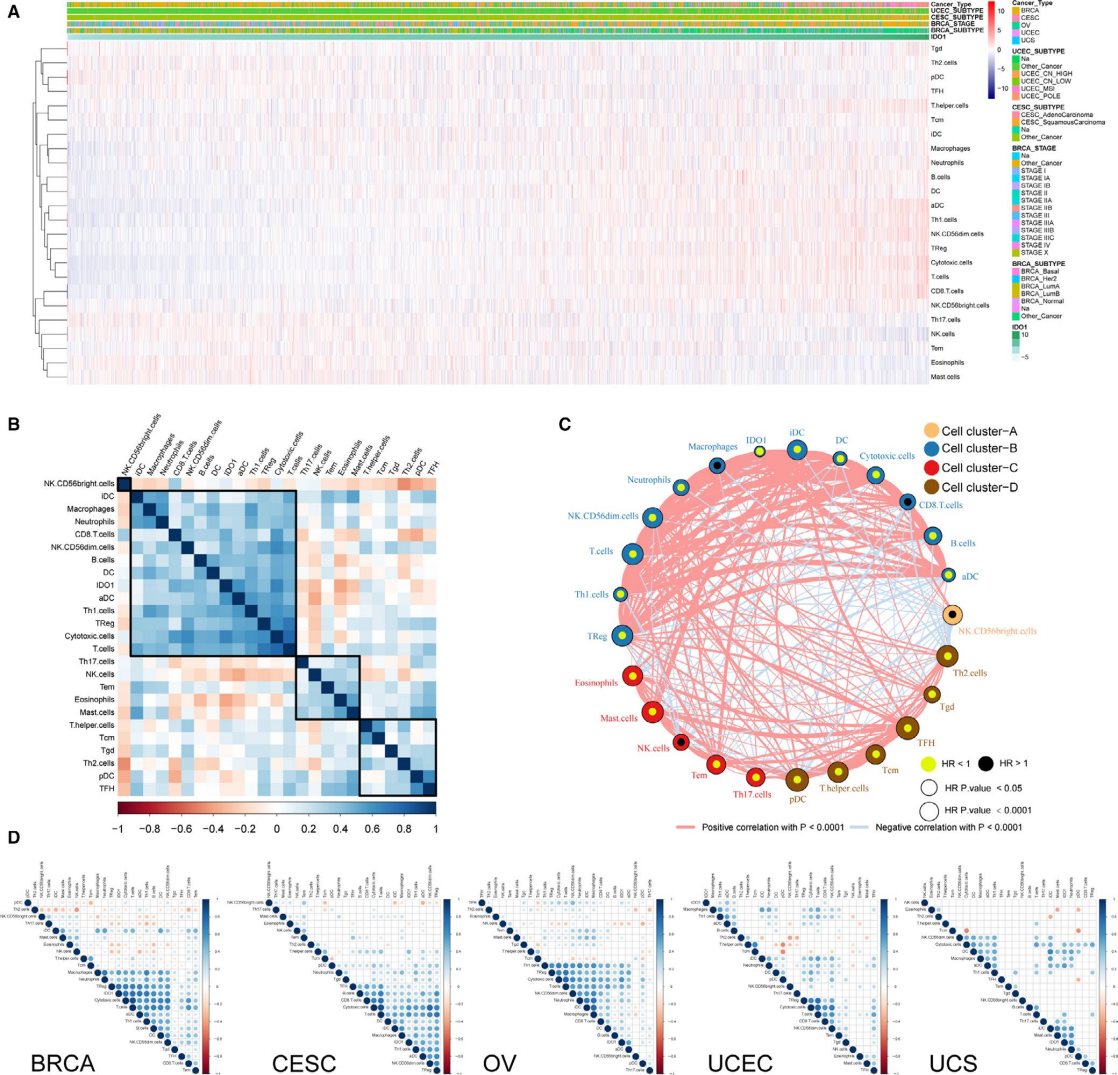
IDO1 expression and tumour‐infiltrating immune cells. A, Heatmap illustrating the association between IDO1 expression and immune cells in gynaecologic and breast cancers microenvironment using single‐sample gene set enrichment analysis scores from 24 immune cell types. B, Correlation clustering of IDO1 and immune cells in the overall Pan‐Gyn based on expression data to classify them into four groups. C, IDO1 and tumour micro environment cells interaction. Cell cluster‐A, NK CD56bright ells; cell cluster‐B, IDO1, aDC, B cells, CD8^+^ T cells, cytotoxic cells, DC, iDC, macrophages, neutrophils, NK CD56dim cells, T cells, Th1 cells, TReg; cell cluster‐C, eosinophils, mast cells, NK cells, Tem, Th17 cells; cell cluster‐D, pDC, T helper cells, Tcm, TFH, Tgd, Th2 cells. The lines connecting each pair of cells represent their cellular interactions. The thickness of each line represents the correlation estimated by Spearman correlation analysis. The dot size of each cell type represents *P*‐value of HR, See also Table S1. Favour for overall survival is indicated in yellow, and risk for overall survival is indicated in black. D, IDO1 and immune cells correlation in each individual cancer types. See also Table S3

Through the single‐sample gene set enrichment analysis, we evaluated the association between IDO1 and tumour‐infiltrating immune cells from transcriptomic data. The landscape of IDO1 and tumour‐infiltrating immune cells correlation is shown in Figure 2A. The correlation clustering classified IDO1 and 24 type of immune cells into four groups, IDO1 was grouped with iDC, Macrophages, Neutrophils, CD8^+^ T cells, NK CD56dim cells, B cells, DC, aDC, Th1 cells, TReg, Cytotoxic cells and T cells. In addition, Th17 cells, NK cells, Tem, eosinophils and mast cells were divided into a group and T helper cells, Tcm, Tgd, Th2 cells and pDC plus TFH were divided into another group. Moreover, NK CD56 bright cells had a unique expression pattern than others (Figure 2B). Furthermore, the IDO1 and immune cells network depicted a comprehensive landscape of tumour‐immune cell interactions, cell lineages and their effects on the overall survival of patients with gynaecologic and breast cancers (Figure 2C, Tables S1 and S2). A strong correlation between IDO1 and aDC in breast and four gynaecologic cancers was discovered. IDO1 was also correlated with CD8^+^ T cells, NK CD56dim cells, Th1 cells and macrophages in more than three different cancers (Figure 2D and Table S3).

### 3.3 IDO1 expression and TMB

**Figure 3:**
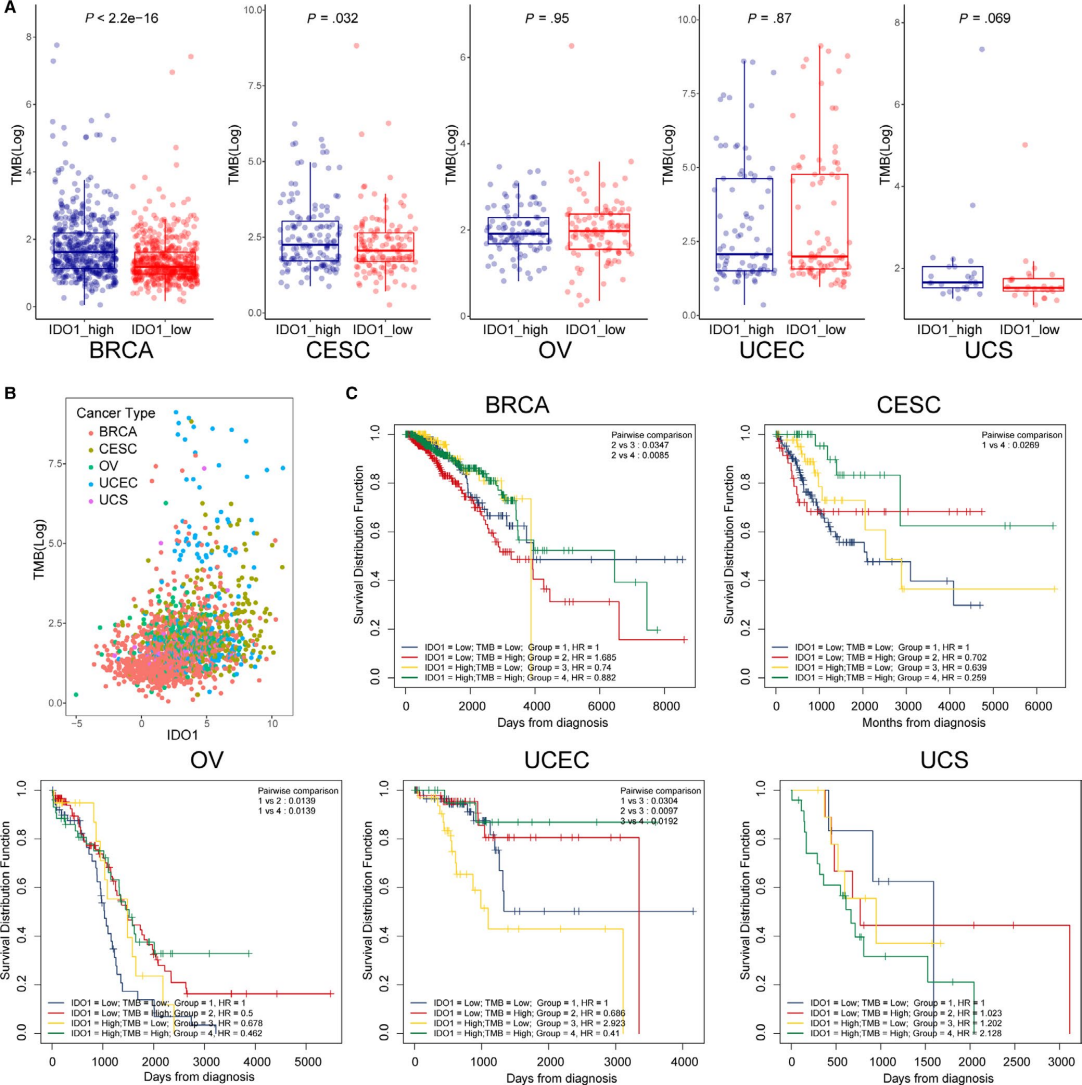
IDO1 expression and tumour mutation burden (TMB) status. A, TMB status with high and low IDO1 expression level. B, Overview of IDO1 expression and TMB status in gynaecologic and breast cancers. C, Overall survival curves using combinations of IDO1 expression level and TMB status. Univariate Cox regression used to estimate HR; log‐rank *P*‐values reported; Bonferroni multiple testing adjustment for pairwise comparisons

Considering tumour mutations can result in immunogenic neo‐antigens, which have also been correlated with responsiveness to a more effective response to immune therapy, we analysed IDO1 expression with TMB status and grouped patients into IDO1‐high and IDO1‐low and analysed their TMB status. As shown in Figure 3A, IDO1 had a higher expression level in TMB‐high group in BRCA and CESC. In addition, UCEC contained a higher TMB level and IDO1 expression than other four female cancers (Figure 3B). It suggests that IDO1 inhibitor may be more effective in TMB‐high patients. Then, we combined group analysis with IDO1 and TMB, and it showed that patients with low IDO1 expression and high TMB have the worse OS than IDO‐high/TMB‐low (*P* = .0347) and IDO‐high/TMB‐high (*P* = .0085) groups in BRCA. In CESC, IDO1‐low/TMB‐low group has the worse OS than IDO1‐high/TMB‐high group (*P* = .0269). In OV, IDO1‐low/TMB‐low group has the worse OS than IDO‐low/TMB‐high (*P* = .0139) and IDO‐high/TMB‐high (*P* = .0139) groups. In UCEC, IDO1‐high/TMB‐low group have the worse OS than other three groups and there was no statistic difference in UCS (Figure 3C). Taken together, these data suggest that both IDO1‐ and TMB‐high group may have a better outcome.

### 3.4 IDO1‐grouped hallmarks and pathway

**Figure 4:**
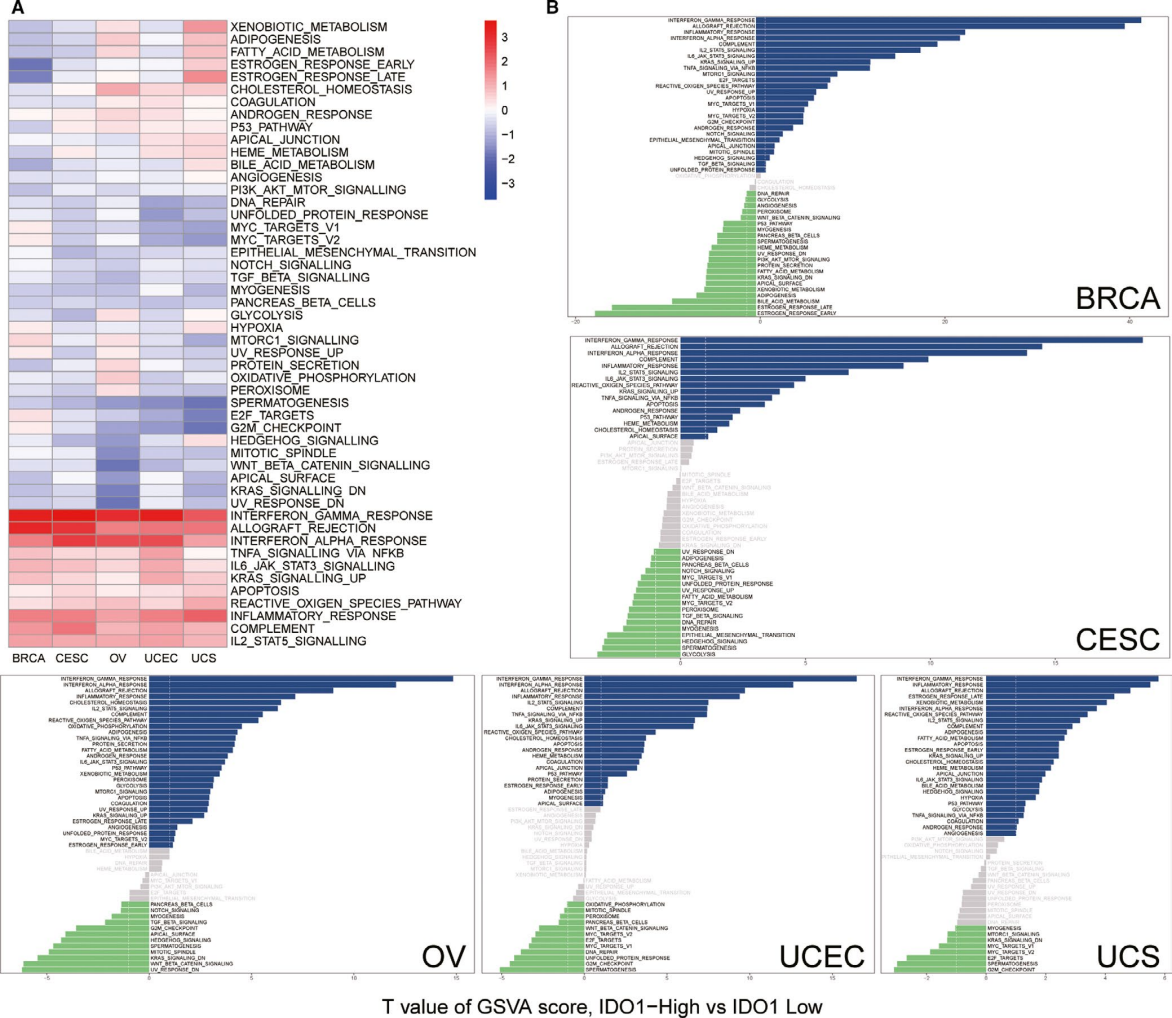
IDO1‐grouped hallmarks and pathway. A, Heatmap of GSVA of IDO1 in Pan‐Gyn. B, GSVA of each female cancer grouped by IDO1 high and IDO1 low

Hallmarks significantly correlated with IDO1 expression were screened out in TCGA (BRCA, CESC, OV, UCEC and UCS) data sets. These hallmarks were further analysed by GSVA (R packages). IDO1 was divided into two groups: IDO‐low (the lowest 30% expression of IDO1) and IDO1‐high (the highest 30% expression of IDO1). We found that these hallmarks with higher IDO1 level in five female cancers were mainly involved in immune‐related hallmarks, including interferon gamma response, allograft rejection and interferon alpha response, TNFa signalling via NF‐kB, IL6‐Jak‐Stat3 signalling, Kras signalling up, apoptosis, reactive oxygen species pathway, inflammatory response, complement and IL2‐Stat5 signalling (Figure 4A). Moreover, spermatogenesis had an inverse correlation with IDO1 (Figure 4A). The unique hallmarks of each cancer are shown in Figure 4B. Interestingly, IDO1 showed an inverse correlation with oestrogen response in breast cancer, while the positive correlation in uterine carcinoma. P53 pathway also showed a negative relation in breast cancer, while a positive relation in other four gynaecologic cancers. However, E2F‐targets, MYC‐targets‐V2, G2M‐checkpoint and mitotic spindle have the opposite trend. Taken together, our analysis suggests that IDO1 in different tumours have some special hallmarks, but they also have some common features around immune response.

### 3.5 IDO1‐related genes and pathway

**Figure 5:**
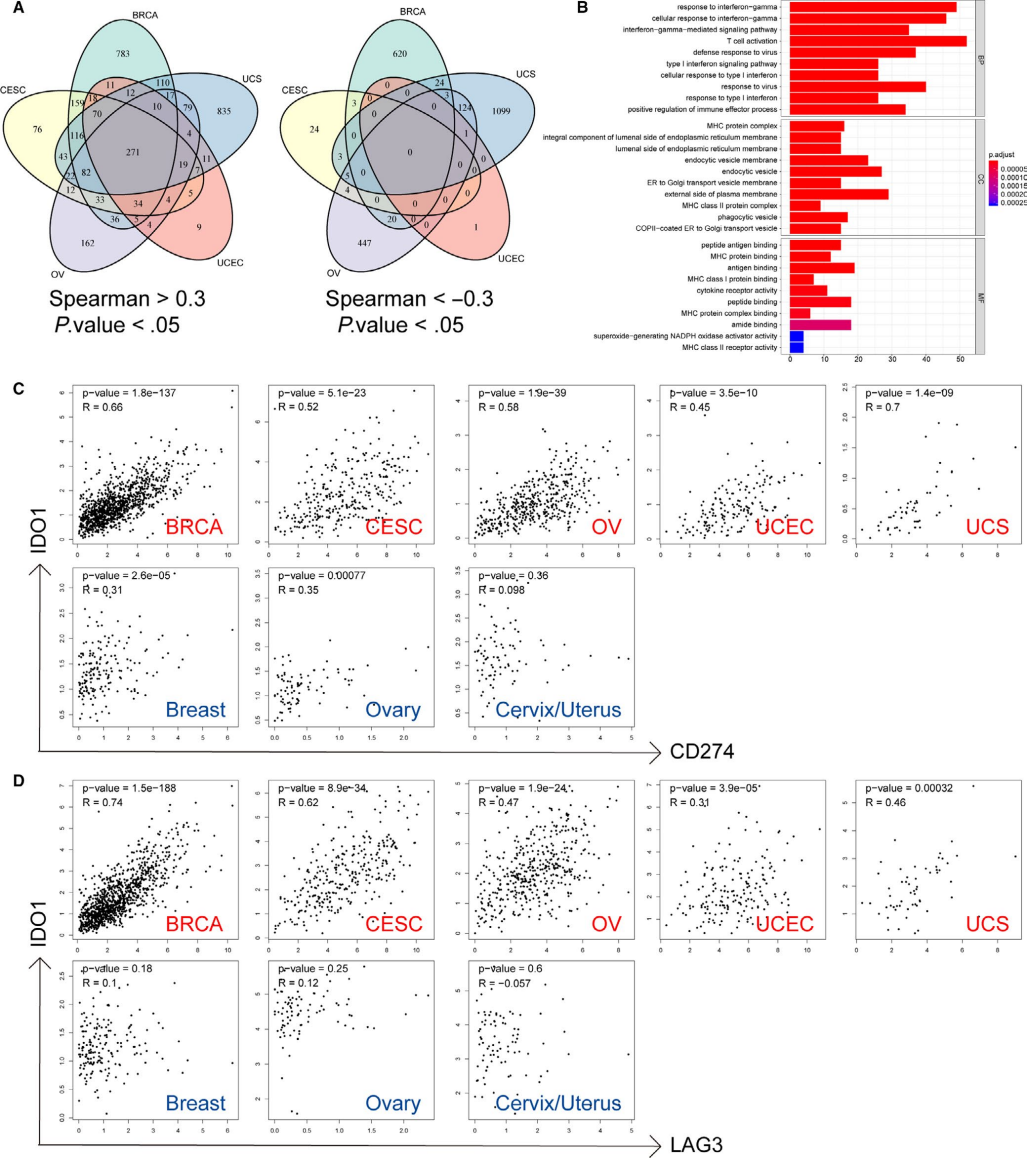
IDO1‐related genes and pathway. A, Venn diagram of IDO1 positively correlated genes in Pan‐Gyn. B, GO enrichment analysis on 271 positively correlated genes in Pan‐Gyn. C, Dot plots of correlations between IDO1 and CD274 (PD‐L1) in gynaecologic and breast cancers and their corresponding normal tissues from GEPIA. D, Dot plots of correlations between IDO1 and LAG3 in gynaecologic and breast cancers and their corresponding normal tissues from GEPIA

To further investigate the role of IDO1 in gynaecologic and breast cancers, we evaluated its co‐expression related genes. We found IDO1 expression was positively correlated (Spearman *R* > .3) with 271 genes in four gynaecologic cancers plus breast cancer, while no negatively correlated genes (Spearman *R* < −.3) appeared simultaneously in those cancers (Figure 5A). Those positively correlated genes were enriched in activation of immune response, such as IFN‐γ response, MHC protein complex and peptide antigen binding (Figure 5B and Table S4). Interestingly, we found that PD‐L1 and LAG3 were highly positive related to IDO1 in gynaecologic and breast cancers, but their correlation was reduced in normal breast tissue, ovary and uterus/cervix (Figure 5C,D). It suggests that IDO1, PD‐L1 and LAG3 were partially regulated by the same mechanism or inducer in tumour.

## 4 DISCUSSION

The immune escape function of IDO1 in cancer was first reported in 2002 by Friberg et al.^19^ Currently, more and more studies have demonstrated that IDO1 is associated with immune escape by suppressing T cell activity and enhancing Treg in different tumour types. However, its role in female tumours lacks a detailed report, so we aimed to provide some new insights into understanding of IDO1 in the regulatory mechanism of female tumours and suggest that IDO1 may be a potential therapy target for female. Our study is the most comprehensive study characterizing the expression pattern of IDO1 together with its related genetic features and prognostic values in gynaecologic and breast cancers.

We revealed that IDO1 was significantly up‐regulated in all of the female tumours according to TCGA database. According to breast cancer PAM50 classification scheme, IDO1 expression was higher in tumours of basal than other subtypes and shown better survival prognosis to BRCA and OV. Since our study found that elevated IDO1 is associated with a better prognosis for breast and ovarian cancer, there have been several reports correlating IDO1 expression with poor patient prognosis, including those diagnosed with colorectal cancer,^20^ prostate cancer,^21^ acute myeloid leukaemia,^22^ glioblastoma,^23^ non‐small‐cell lung cancer^24^ and oesophageal cancer.^25^ To our surprise, ovarian carcinoma^26^ and endometrial cancer^27^ also included. Other studies indicated reversely that high IDO1 expression levels are correlated with a favourable survival, which include renal cell carcinoma,^28^ breast cancer^29^ and hepatocellular carcinoma.^30^ The complexity of clinical outcome associated with IDO1 level could be explained by the following reasons. Firstly, the IDO1 expression level in TCGA database was only detected by next gene sequencing using the whole RNA extracted from tumours which contained both tumour cells (TCs) and tumour‐infiltrating cells (TILs). Actually, IDO1 is expressed in two compartments, TCs and TILs, respectively, and the tumour microenvironment may be changed by their interaction, leading to variant outcomes of tumour prognosis. Secondly, environment tryptophan deprivation has been reported to also decrease tumour cell proliferation.^31^, ^32^ Thirdly, IFN‐γ, mainly produced by activated infiltrating T cells, has been reported to be associated with a better survival and IDO1 could be induced by it.^29^, ^33^, ^34^ Furthermore, IFN‐γ is secreted during the anti‐tumour immune response. IDO1 level could therefore also be a marker of an ongoing anti‐tumour immune response. Additionally, our study is not the only one to report a favourable prognostic impact of IDO1 expression in cancer cells. Riesenberg et al^28^ investigated that the immunogenic RCC tumours especially benefit from the presence of IDO1. Their IHC results showed that IDO1 is nearly expressed in endothelial cells of predominantly newly formed blood vessels. The ratio of IDO1 positive vessels inversely correlated with the proliferating tumour cells. Therefore, IDO1 in endothelial cells might limit the influx of tryptophan from the blood to the tumour or generate tumour‐toxic metabolites, thus restricting tumour growth and contributing to survival. Ishio et al^30^ reported that IDO1 mRNA correlated significantly with gene expression of IFN‐γ, TNF‐α and IL‐1β. The recurrence‐free survival rate of IDO1‐positive HCC patients was significantly higher than that of IDO1‐negative HCC patients. They also found the IDO1 inhibitor significantly reduced such cytotoxicity of PBMC against certain HCC cell lines. These findings raise the possibility that IDO1 mRNA is expressed in TILs of tumour tissue due to the presence of these cytokines, which might be produced by activated tumour‐infiltrating cells. Li et al^35^ confirmed those opinions, and their survival analyses showed that increased tumour IDO1 and CD8^+^ T cell infiltration were significantly associated with superior overall survival. Their data showed that tumours with a high level of IDO1 expression were more likely accompanied by abundant CD8^+^ T cell infiltration. Jacquemier et al^29^ also showed a positive correlation between IDO1 and TILs in breast cancer. In their study, IDO1 was the most significant marker associated with better outcome. Moreover, other studies showed that IDO1 was also expressed in various non‐tumour cells in the tumour microenvironment, such as dendritic cells, fibroblasts, endothelial cells, eosinophils and macrophages which may increase the contradictory role of IDO1 with clinical outcome.

Although there is a difference between these tumours regarding their correlation with IDO1 expression level and clinical outcome, both investigations appear to have a positive correlation between the presence of tumour‐infiltrating T cells and increased IDO1 expression. Consistent with previous studies, we found a strong correlation between IDO1 and immune cell populations especially for dendritic cells and T cells. Studies reported that IDO1 could be induced by IFN‐γ which mainly produced by activated infiltrating T cells and seems to be a key player in the innate immune system.^32^ Our results also showed that IDO1 was significantly correlated with T cells and IFN‐γ. Furthermore, our results also showed the correlation of IDO1 and antigen‐presenting cells (APCs), like dendritic cells and macrophages, and other studies investigated those APCs could mediate immune response via IDO1 through cell cycle arrest in T cells. Evidence has shown that IFN‐γ and tumour infiltration by activated CD8^+^ cytotoxic T lymphocytes correlate with better survival.^36^ Due to the positive correlation between IDO1 and TILs/ IFN‐γ, IDO1 can be subsequently related to a better prognosis.

Since tumours with a higher mutation burden have been hypothesized to have more chance to produce neoantigens and these neoantigens can be recognized by the immune system. To learn more about the potential relation between tumour mutation and IDO1, we investigated the association between IDO1 and TMB, and found that IDO1 was significantly correlated with TMB in BRCA and CESC. We also observed that high IDO1 expression and high TMB had a better clinical outcome than other IDO1/TMB expression patterns in female cancer except uterine carcinosarcoma. This point may be contrary to the routine study and conclusions, in which patients with high TMB usually display poor clinical outcome, but nothing is absolute. There is another view that TMB‐high cancer may exhibit an active tumour immune microenvironment, which is due to the recognition of a large number of tumour‐specific neoantigens. The tumour‐specific neoantigens produced by a TMB‐high tumour are presented on APCs and stimulate T cell activation, leading to an active Th1/CTL microenvironment which exhibits a better outcome for patients. Not only in the present study, but also in Liu et al^37^ study, they showed that BLCA exhibited a correlation between higher TMB and improved overall survival, which indicated the existence of inter‐tumour heterogeneity of TMB as a prognostic biomarker. Moreover, we should take MSI status into consideration. The majority of MSI‐high tumours were TMB‐high; however, only few of TMB‐high tumours were MSI‐high.^37^ In the previous studies,^38^ MSI‐high tumours have shown a better prognosis than microsatellite stable (MSS), where their higher mutational load may contribute to better survival. Therefore, the MSI and immune infiltrating status increased complexity and diversity between TMB status and prognosis.

Our analysis also found the correlation between increasing IDO1 levels with other immune checkpoints, including PD‐L1, LAG3, CD86, IRFs and HLAs in female cancers. What is more, we found that immune‐related genes, such as PD‐L1 and LAG3, were highly positive related to IDO1 in gynaecologic cancers when comparing with their corresponding normal tissues. PD‐L1 and LAG3 can be induced by IFN‐γ, the same as IDO1, and this may partially explain their positive correlation in cancer samples. Up‐regulation of immune checkpoint proteins has been linked to cancer immune escape, but in our study, they showed a better prognosis and we explained the mechanism of IDO1 in the above. PD‐L1 also has the contradictory phenomenon. Previous studies indicated the correlations between PD‐L1 and reduced prognosis, but some other studies^38^, ^39^, ^40^ found that PD‐L1 expression is paradoxically associated with improved patients' survival. Lee et al^38^ investigated that PD‐L1, LAG3 and IDO1 expressions in tumour‐infiltrating immune cells were significantly associated with a better disease‐free survival. This is similar to the findings of Webb et al,^39^ where IDO1 expression in tumour‐infiltrating immune cells was associated with PD‐L1, LAG3 and CTLA4, resulting in a positive correlation with survival. Those findings are also consistent with the concept of adaptive resistance, wherein activated T cells trigger negative feedback mechanisms in the tumour microenvironment, resulting in an immune equilibrium. One hypothesis is that patients undergo standard treatment will re‐activated these responses, resulting in improved tumour control and a favourable prognosis.

Our results indicated that IDO1 participated in tumour immune process. These findings may expand our outlook of potential anti‐IDO1 treatments. Preclinical and clinical trials have demonstrated a trend of multi‐immunotherapeutic agent combination showed a greater survival benefit over single‐agent approaches. It is possible to combine strategies targeting multiple immune checkpoints. Different studies reported that combined with other immunotherapeutic agent, such as CTLA‐4 or PD‐1/PD‐L1 inhibitors, this objective response rates range from 10% to 57% among different tumour types. These findings lead to promising opportunities for the combined synergistic treatment of female cancers.

In conclusion, the involvement of IDO1 in different cancers appears to be highly complex. Despite this complexity, this is the first study exploring the expression pattern, clinical outcome, tumour‐infiltrating, TMB and biological processes in gynaecologic and breast cancers. Our results revealed the role of the IDO1 in tumour progression and immune responses that may lead to the development of IDO1 targeting therapy for assessing the efficacy and receptiveness in female cancer treatment.

## CONFLICT OF INTEREST

All authors declare no conflicts of interest.

## AUTHOR CONTRIBUTION

Xu Feng, Ranran Tang and Runjie Zhang conceived the study. Xu Feng downloaded the data. Xu Feng drafted the manuscript and performed the analysis. Huan Wang, Zhaodong Ji, Yang Shao, Shuoer Wang and Jiao Meng participated in analysis design. Yun Gu, Tianying Zhong and Jiao Meng contributed to drafting the manuscript, interpreting data and coordinating the study. All authors read and approved the final manuscript.

## ETHICAL APPROVAL

All analyses were based on previously published TCGA data; thus, no ethical approval and patient consent are required.

## Supporting information

Figure S1

Figure S2

Table S1

Table S2

Table S3

Table S4

## Data Availability

The data used to support the findings of this study are available from the corresponding author upon request.
