# Supplementary figures and images for: A comprehensive analysis of IDO1 expression with tumour‐infiltrating immune cells and mutation burden in gynaecologic and breast cancers
Source: J Cell Mol Med. 2020 Mar 30;24(9):5238–48. doi: 10.1111/jcmm.15176 (PMC7205837; doi:10.1111/jcmm.15176)

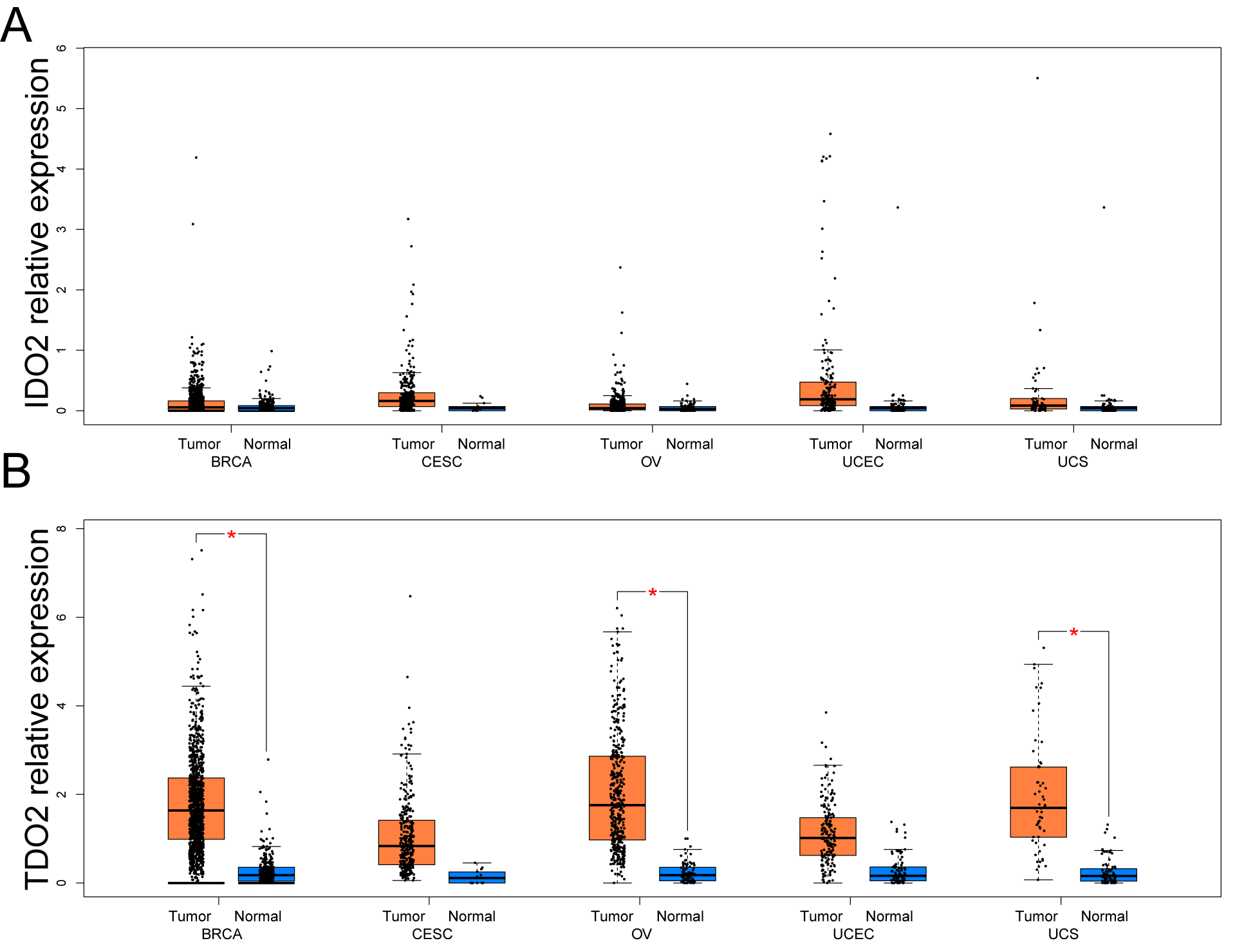

Supplement: Supplementary file 1 — Figure S1 [file JCMM-24-5238-s001.tif]

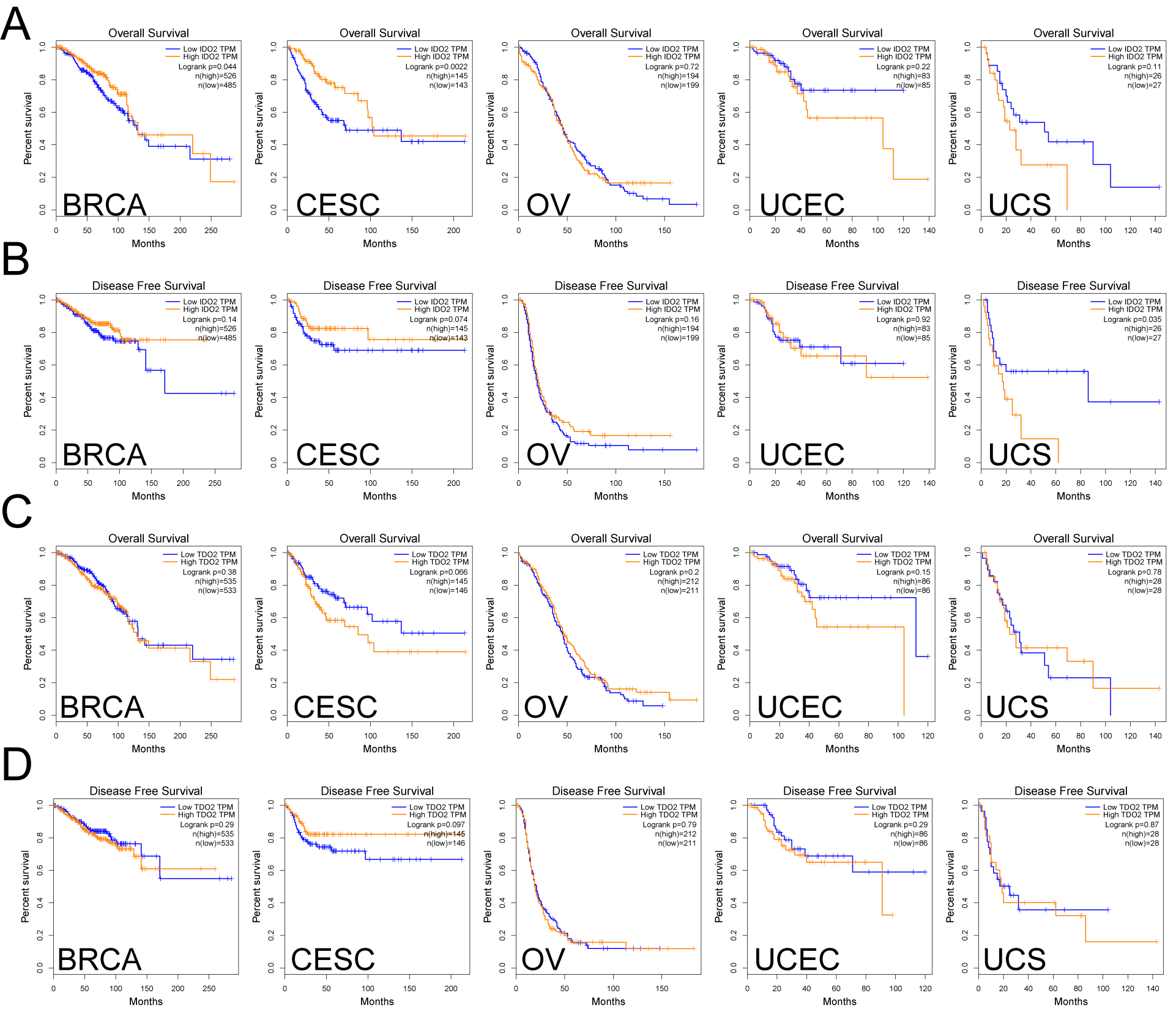

Supplement: Supplementary file 2 — Figure S2 [file JCMM-24-5238-s002.tif]
